# Targeting Glioblastoma Stem Cells to Overcome Chemoresistance: An Overview of Current Therapeutic Strategies

**DOI:** 10.3390/biomedicines10061308

**Published:** 2022-06-02

**Authors:** Hyunkoo Kang, Haksoo Lee, Dahye Kim, Byeongsoo Kim, JiHoon Kang, Hae Yu Kim, HyeSook Youn, BuHyun Youn

**Affiliations:** 1 Department of Integrated Biological Science, Pusan National University, Busan 46241, Korea; kanghk94@gmail.com (H.K.); hs950303@gmail.com (H.L.); hye10280@gmail.com (D.K.); djsej18@gmail.com (B.K.); 2 Department of Hematology and Medical Oncology, Winship Cancer Institute of Emory, Emory University School of Medicine, Atlanta, GA 30322, USA; jhkang4293@gmail.com; 3 Department of Neurosurgery, Haeundae Paik Hospital, Inje University College of Medicine, Busan 48108, Korea; hykim0803@paik.ac.kr; 4 Department of Integrative Bioscience and Biotechnology, Sejong University, Seoul 05006, Korea; hsyoun@sejong.ac.kr; 5 Department of Biological Sciences, Pusan National University, Busan 46241, Korea

**Keywords:** glioblastoma, chemoresistance, cancer stem cells, temozolomide

## Abstract

Glioblastoma (GBM) is the most malignant primary brain tumor. The current standard approach in GBM is surgery, followed by treatment with radiation and temozolomide (TMZ); however, GBM is highly resistant to current therapies, and the standard of care has not been revised over the last two decades, indicating an unmet need for new therapies. GBM stem cells (GSCs) are a major cause of chemoresistance due to their ability to confer heterogeneity and tumorigenic capacity. To improve patient outcomes and survival, it is necessary to understand the properties and mechanisms underlying GSC chemoresistance. In this review, we describe the current knowledge on various resistance mechanisms of GBM to therapeutic agents, with a special focus on TMZ, and summarize the recent findings on the intrinsic and extrinsic mechanisms of chemoresistance in GSCs. We also discuss novel therapeutic strategies, including molecular targeting, autophagy inhibition, oncolytic viral therapy, drug repositioning, and targeting of GSC niches, to eliminate GSCs, from basic research findings to ongoing clinical trials. Although the development of effective therapies for GBM is still challenging, this review provides a better understanding of GSCs and offers future directions for successful GBM therapy.

## 1. Introduction

Glioblastoma (GBM) is one of the most recalcitrant tumors among all malignant solid tumors [1]. Patients with GBM have a poor prognosis, with a 5-year survival rate of 6.8% and a median overall survival of approximately 12–15 months [2]. The standard therapy for patients with GBM is surgical resection, followed by radiotherapy and chemotherapy with the alkylating antineoplastic agent, temozolomide (TMZ) [3]. However, considering the difficulty in distinguishing the anatomical borders of GBM and its extreme infiltrative growth, complete surgical resection is practically impossible [4]. In addition, GBM is highly resistant to conventional therapies because of its high heterogeneity resulting from clonal evolution due to genome instability and the differentiation of GBM stem cells (GSCs) [5]. Accordingly, the majority of patients with GBM experience tumor recurrence, and recurrent tumors commonly show a poor response to standard therapy [6]. Despite advancements in the understanding of GBM molecular diagnosis and ongoing trials on targeted therapies, standard therapy has not advanced over the past two decades [7].

TMZ is an oral alkylating agent used for the first-line treatment of GBM and anaplastic astrocytoma since its U.S. Food and Drug Administration (FDA) approval in 2005 [8]. TMZ induces cytotoxicity by adding a methyl group to the N^7^ and O^6^ positions of guanine residues and N^3^ position of adenine residue during DNA replication. Alkylation of guanine or adenine results in mismatched base pairing that induces DNA strand breakage and cell cycle arrest at the G_2_/M phase, thereby leading to cell apoptosis [9]. As TMZ is the only chemotherapeutic agent currently used in patients with GBM, a strategy for overcoming TMZ resistance is urgently needed. The main contributor to TMZ resistance is O^6^-methylguanine-DNA methyltransferase (MGMT), which repairs the mutagenic DNA adduct, O^6^-methylguanine, back to guanine. Although methylation at the guanine O^6^ position is the least frequent adduct formed by TMZ, MGMT efficiently prevents the TMZ-induced formation of lethal DNA cross-links [10]. Nevertheless, approximately half of the patients with GBM harbor the MGMT promoter methylation, which acts as a prognostic indicator of whether a patient benefits from TMZ treatment [11]. However, some patients with GBM suffer from TMZ resistance despite their low MGMT activity, indicating that MGMT is not the only determinant of TMZ resistance.

Cancer stem cells represent a population of tumor cells that are capable of self-renewal and differentiation [12]. GSCs are also characterized by their ability to form tumorspheres with sustained proliferation in vitro and form tumors upon serial transplantation in vivo. Specifically, they contribute to high levels of intratumoral cellular heterogeneity and plasticity in GBM, thereby causing radioresistance and chemoresistance to induce tumor recurrence [13,14]. The stem state of GSCs is not static but plastic; therefore, interconversion between GSC and non-GSC states can occur according to various factors, including nutrient deprivation, hypoxia, radiation and chemotherapeutic treatment [13,15]. Targeting GSCs in combination with conventional therapies could be a promising therapeutic strategy for eradicating GBM, and several putative GSC surface markers, such as CD133, CD15, and CD44, and GSC transcription factors, such as SRY-box transcription factor 2 (SOX2), octamer-binding transcription factor 4 (OCT4), and NANOG, have been discovered [16]. However, the known GSC markers are neither completely sensitive nor specific to the GSC population [13] and, hence, have not been used in clinical practice, despite numerous strategies to target GBM stemness [17].

This review summarizes the findings of recent studies about various mechanisms of chemoresistance of GSCs, with a special focus on the resistance mechanism to TMZ, the first-line drug for GBM treatment. Additionally, we have described recent therapeutic strategies to directly or indirectly target GSCs and summarize the ongoing clinical trials targeting GSCs. Although GBM remains incurable and an effective therapeutic strategy has not yet been established, understanding GSCs will provide new directions for the treatment of GBM.

## 2. Mechanisms of Chemoresistance in GSCs

**Figure 1:**
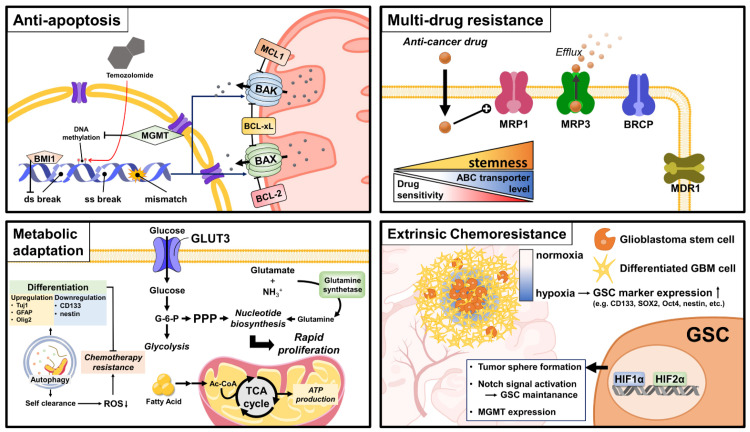
Comprehensive schematic diagram of various mechanisms of chemoresistance in GSCs. GSC-specific pathways related to anti-apoptosis, multi-drug resistance, metabolic adaptation, and extrinsic resistant mechanisms are key for GSC chemoresistance and maintenance.

Based on recent findings, we describe various resistance mechanisms of GBM to therapeutic agents, particularly TMZ, including DNA repair systems, anti-apoptosis, multidrug resistance (MDR), metabolic rewiring, autophagy regulation, and extrinsic resistant mechanisms (Figure 1).

### 2.1. DNA Repair Systems

MGMT expression considerably increases the resistance to alkylating agents in GSCs [18]. GSCs express significantly higher levels of MGMT than stable glioma cell lines [19]. Similarly, MGMT levels in CD133-positive cells are higher than those in autologous CD133-negative cells [20]. Moreover, irrespective of MGMT-dependent repair, CD133-positive GBM cells preferentially activate the checkpoint kinases 1 (Chk1) and 2 (Chk2), the key regulators of DNA checkpoint signaling, to repair DNA damage more effectively than autologous CD133-negative GBM cells [21]. CD133-positive cells are predominantly present in the inner tumor mass of GBM to avoid chemotherapeutic treatment. Accordingly, MGMT is more highly expressed in the inner core than in the peripheral area of GBM tumor mass [22]. In contrast, GSCs are not uniformly resistant to TMZ, and the methylation status cannot precisely explain the response to TMZ [23]. In addition, TMZ treatment at 500 μM induced cell death (25%) in neural stem cells (NSCs), while GSCs were rarely affected, even though GSCs and NSCs have similar levels of MGMT, indicating the possible existence of an alternative resistance mechanism [19]. These studies suggest that GSCs are resistant to alkylating agents not only because of the high expression of MGMT, but also because of a mechanism independent of MGMT-related therapeutic resistance.

Polycomb-group proteins are transcriptional regulators that mediate epigenetic silencing and multicellular development [24]. BMI1, a member of the polycomb group complex 1, is recruited to DNA damage sites and plays a role in DNA double-strand break repair. BMI1 is highly enriched in CD133-positive GBM cells and is required for maintaining GSC self-renewal and intracranial GBM tumor formation [25]. Moreover, BMI1 is preferentially copurified with proteins involved in non-homologous end joining, including DNA-PK, poly (ADP-ribose) polymerase-1, hnRNP U, and histone H1, in CD133-positive GBM cells [26]. In contrast, a separate study showed that there was no difference in the DNA repair mechanisms between GSCs and differentiated cells, but GSCs showed an elongation of all phases of the cell cycle with enhanced basal activation of checkpoint proteins [27].

### 2.2. Anti-Apoptosis

Treatment with TMZ, the first-line drug for GBM, induces prolonged p53- and p21(Waf1/Cip1)-associated cell cycle (G_2_/M) arrest [28]. Cytoprotective anti-apoptotic mechanisms are likely activated in GSCs. TMZ-resistant GSCs show higher expression levels of several anti-apoptotic genes, such as B-cell lymphoma-2 (BCL-2), BCL2 like 1 (BCL2L1), and MCL1, compared to differentiated cell lines [29]. Notably, the critical anti-apoptotic protein, BCL-xL/BCL2L1, is highly expressed in GSCs and glioblastoma stem-like cells (GSLCs) cultured using the tumorsphere method in comparison to differentiated cells and prevents intrinsic apoptosis induced by alkylating agents [30]. In addition, the inhibition of BCL-extra-large (BCL-xL)/BCL2L1 decreases the size and number of GSLCs. GSC-enriched protein, BMI1, is required to prevent p53-mediated apoptosis, suggesting that GSCs preferentially avoid TMZ-induced apoptosis, conferring therapeutic resistance to GBM [31]. These data suggest that GSC-specific anti-apoptotic mechanisms contribute to chemoresistance in GBM.

### 2.3. Multidrug Resistance

MDR is a generic term for a cross-resistant phenotype against various unrelated drugs in cancer cells. There are diverse mechanisms associated with MDR, including anti-apoptosis, detoxification, prevention of drug uptake, activation of drug efflux, and DNA repair systems [32]. The ATP-binding cassette (ABC) transporter family, which pumps drugs out of cells, is one of the most studied gene families related to MDR [33]. Likewise, the ABC transporters, MRP1 (ABCC1), MRP3 (ABCC3), and MRP4 (ABCC4), are highly expressed in glioma cells [34]. Interestingly, ABC transporters are predominantly expressed in cancer stem cells [35], and GSCs exhibit high expression levels of MRP1, MRP3, and MDR1 (ABCB1) [34]. The expression levels of MRP1 and MDR1 in CD133-positive GSCs were higher than those in differentiated tumor cells [36]. GSLCs derived from the U87MG cell line also showed high expression of MDR1, indicating that GSCs display intrinsic MDR [37]. In addition, treatment with the chemotherapeutic medications, etoposide and TMZ, further increased the expression levels of MRP1 in CD133-positive U251 GSLCs, presumably because of the plasticity of cancer stem cells [37,38]. In the same study, knockdown of livin, an anti-apoptotic protein, suppressed cell proliferation with a decrease in MRP1 expression and an increase in MRP3 expression in GSLCs [38]. Taken together, ABC transporters, especially MRP1, MRP3, and MDR1, are highly expressed in GSCs to prevent the drug entry, and each protein may play a different role depending on its cellular context.

Breast cancer resistance protein (BCRP, also known as ABCG2) is related to the grade and chemoresistance of glioma [39]. The expression levels of ABCG2 are significantly higher in high-grade gliomas (grades III and IV) than in low-grade gliomas (grades I and II). Interestingly, immunostaining of ABCG2 showed that 100% of the CD133-positive glioma stem cells were ABCG2-positive, whereas no CD133-negative fraction expressed ABCG2. In addition, treatment with nicardipine, an ABCG2 competitive inhibitor, sensitized CD133-positive glioma stem cells to mitoxantrone, whereas no synergistic effect was observed in CD133-negative tumor cells. Likewise, inhibition of ABCG2 by melatonin-induced promoter methylation showed synergistic toxic effects with the chemotherapeutic agents, TMZ, doxorubicin, and mitoxantrone [40]. ABCG2 expression is increased by the overexpression of OCT4, which regulates GSC properties, and OCT4-overexpressed U251 GBM cells are resistant to the chemotherapeutic agents, doxorubicin, carboplatin, and VP16 [41]. These data suggest that the expression levels of ABC transporters are strongly related to the stem phenotype of GSCs and contribute to chemoresistance.

### 2.4. Metabolism

Metabolic dysregulation of cancer cells is a hallmark of cancer [42,43,44]. As GSCs must survive harsh conditions, characterized by hypoxia and low nutrient supply, they have high bioenergetic needs to maintain their rapid proliferation and stemness [13]. GSCs preferentially take up glucose using the high-affinity glucose transporter 3 (GLUT3) to meet their high metabolic demands [45,46]. Glucose obtained in this manner provides a carbon source for nucleotide biosynthesis to support rapid cell proliferation [47]. Additionally, the expression levels of glutamine synthetase are significantly higher in GSCs than in autologous differentiated cells, and the synthesized glutamine is utilized for de novo purine biosynthesis instead of replenishing the tricarboxylic acid cycle intermediates [48]. These anabolic advantages of GSCs may contribute to their chemoresistant phenotype. In contrast, the resistant clones of GSCs that survived radiotherapy and TMZ treatment showed high expression levels of genes involved in genomic maintenance and DNA repair pathways (SPT16 homolog, zinc finger CCCH-type containing 11A, chromosome 5 open reading frame 24, translocated promoter region, DExH-box helicase 9, and matrin 3), while genes with functions associated with the inhibition of glucose uptake and suppression of insulin/Akt signaling (ectonucleotide pyrophosphatase/phosphodiesterase 2, early growth response 1, ITPR interacting domain containing 2, and protein phosphatase 2 scaffold subunit alpha) were upregulated. Moreover, genes related to lipid catabolism and detoxification of lipid peroxidation products (aldehyde dehydrogenase 3 family member A2, phospholipase D family member 3, and oxysterol binding protein like 8) were upregulated, suggesting that therapy-resistant GSCs may preferentially use fatty acids, rather than glucose, as a major energy source for ATP production [49]. Similarly, slow-cycling GSCs that rely on oxidative phosphorylation and lipid metabolism are more resistant to TMZ than fast-cycling GSCs that mainly utilize glycolysis [50]. Taken together, GSCs require high metabolic flux to maintain their anabolic requirements, and TMZ-resistant GSCs tend to increase fatty acid oxidation, although they are likely context-dependent.

### 2.5. Autophagy

Over the last decade, autophagy was found to play dual roles in both cytoprotection and cell death in GBM [51,52,53]. Accordingly, whether autophagy should be inhibited or induced in GBM treatment is unclear. As autophagy is a self-clearance pathway that maintains basal reactive oxygen species (ROS) levels by removing the dysfunctional organelles and oxidized peroxisomes, it can protect cells from chemotherapeutic agent-induced ROS [54]. Autophagy induction may contribute to maintaining the stemness characteristics of GSCs [55]. For example, in several recent studies, autophagy inhibitors, such as chloroquine (CQ), were found to sensitize GSCs to TMZ [55,56,57]. Moreover, autophagy is activated by the autophagy-associated factors, DNA damage regulated autophagy modulator 1 and p62/sequestosome 1, in migrating and invading GSCs [58]. However, other studies have shown that autophagy induction using mammalian target of rapamycin (mTOR) inhibitors induces anti-proliferative and pro-differentiating effects on GSCs. Autophagy suppresses the self-renewal ability and tumorigenicity of GLSCs by promoting Notch1 degradation [59]. Likewise, curcumin-induced autophagy also suppresses GSLC self-renewal and proliferation by upregulating the differentiation markers, Tuj1, GFAP, Olig2, and βIII-tubulin, and downregulating the GSC markers CD133 and nestin [60]. Autophagy induction promotes GSC differentiation and increases GSC sensitivity to DNA damage [61,62]. Taken together, autophagy may act as a double-edged sword for both the tumor-promoting and tumor-suppressive effects in GSCs.

### 2.6. Extrinsic Chemoresistance

Despite the intrinsic chemoresistant phenotype of GSCs, TMZ can eradicate GSCs in vitro, suggesting that GSC chemoresistance is not solely dependent on its specific gene expression signature [63]. Various extrinsic factors contribute to chemoresistance. One of the most important extrinsic factors is the hypoxic GBM microenvironment. Rapid cell proliferation and tumor growth with erratic tumor neovascularization in GBM leads to a hypoxic tumor microenvironment [64], and GSCs are more likely to suffer from hypoxia as they are mainly located in the inner tumor mass [22]. Hypoxia increases the expression levels of GSC markers and promotes a cancer stem-like phenotype [65]. Hypoxia-response genes, including hypoxia inducible factor (HIF)-2A and vascular endothelial growth factor (VEGF), were highly expressed in GSCs, and the expression of both HIF-1α and HIF-2α was required for tumorsphere formation and stemness maintenance [66]. The acidic microenvironment also promotes tumorsphere formation and the tumorigenic capacity of GSCs with increased expression of HIF2α and GSC markers [67]. A separate study discovered that HIF-1α activates the Notch signaling pathway, which is essential for GSC maintenance [68].

GSC enrichment by extrinsic factors, including hypoxia, contributes significantly to chemoresistance. Stabilization of HIF-1α positively regulates MGMT expression and contributes to TMZ resistance in CD133-positive cells, but not in CD133-negative cells [69]. Clinically relevant doses of TMZ increase the GSC pool by increasing the expression levels of GSC markers, including CD133, SOX2, Oct4, and nestin, in non-GSC subpopulations, suggesting that chemotherapy-induced GSC amplification is a result of phenotypic conversion from non-GSCs to GSCs [15]. Moreover, extracellular vesicles derived from hypoxic GSCs highly promote TMZ resistance in GBM by delivering miR-30b-3p transcriptionally induced by HIF-1α and signal transducer and activator of transcription 3 (STAT3) [70]. Therefore, the hypoxic microenvironment not only induces metabolic alteration toward glycolysis, but also contributes to DNA repair and stemness of GSCs, resulting in increased chemoresistance.

## 3. Strategies Targeting GSCs

**Figure 2:**
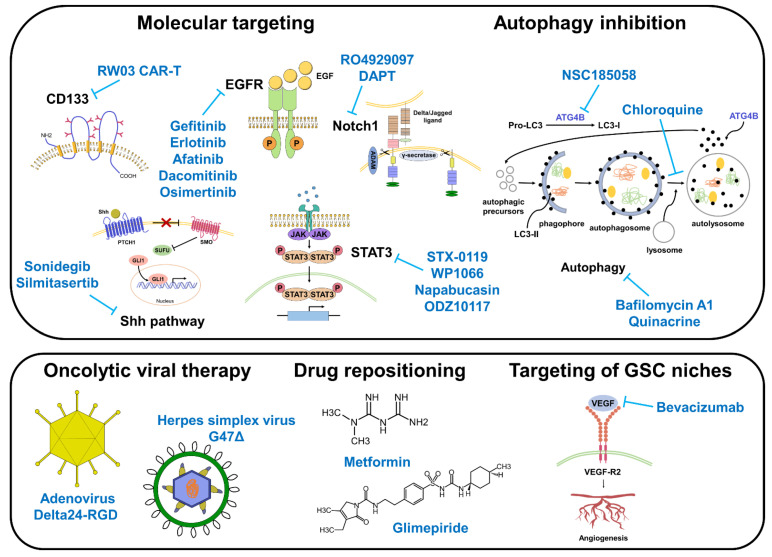
Therapeutic strategies targeting GSCs. Various approaches exist for targeting GSCs, including molecular targeting, autophagy inhibition, oncolytic viral therapy, drug repositioning, and targeting of GSC niches.

Targeting cancer stem cells is the most promising therapeutic strategy, especially for GBM, considering its chemoresistant phenotype and significantly high relapse rate. Although its clinical application remains challenging, several basic research and clinical trials that selectively target GSCs have been reported. In particular, based on the discovered characteristics of GSCs, GSC-targeting drugs have been developed using several therapeutic modalities, including molecular targeting, autophagy inhibition, oncolytic viral therapy, drug repositioning, and indirect targeting of GSC niches (Table 1, Figure 2).

### 3.1. Targeting GSC Markers and Related Signaling Pathways

Several GSC markers have been identified as useful targets for defining the GSC population. GSC markers and related signaling pathways have been implicated in GSC maintenance, stemness characteristics, and resistance to conventional therapies. In this regard, several studies from basic to clinical levels, have selectively targeted GSC markers, including CD133, epidermal growth factor receptor (EGFR), Notch1, sonic hedgehog (Shh), and STAT3, as well as related signaling pathways.

CD133 is one of the most well-characterized cell surface markers used for GSC isolation. As CD133 is a cell surface protein, it can be used as a target for antibody-based therapy. A recent study suggested three immunotherapeutic methods targeting CD133-positive cells: use of synthetic monoclonal antibody, dual-antigen T cell engager, and chimeric antigen receptor (CAR) T cell. The anti-CD133 synthetic antibody, RW03, significantly reduces the self-renewal ability, while barely changing the proliferative capacity of GSCs in vitro. The dual-antigen T cell engager that recognizes CD3 and CD133 on T cells and GBM cells, respectively, effectively eliminates GSCs in the presence of cytotoxic T cells in vivo. CD133-targeting CAR T cells also exhibit high anti-tumor activity in vivo without inducing acute toxicity in normal hematopoietic stem cells that express CD133 [71]. Considering that most immunotherapeutic strategies targeting GSCs focus on antigen-specific immunotherapy, CAR T cell therapy could be a promising strategy to target GSCs [88]. In addition, CD133-positive cells can be selectively targeted and diminished by photothermal therapy. GBM cells were incubated with single-walled carbon nanotubes conjugated with a CD133 monoclonal antibody, followed by near-infrared laser exposure. The tumorigenicity of CD133-positive cells both in vitro and in vivo was significantly inhibited, indicating their potential for use as photothermal therapeutic agents to effectively target GSCs [89].

EGFR is a transmembrane receptor tyrosine kinase that is overexpressed in GSCs to promote self-renewal and tumorigenicity. In particular, EGFR variant III (EGFRvIII) is the most common mutation in GBM and is detectable in 25–33% of patients with GBM [90]. A bispecific antibody targeting EGFRvIII and CD133 specifically eliminates EGFRvIII/CD133-positive cells and shows higher cytotoxicity in GSCs compared to the antibody against either EGFRvIII or CD133 [91]. To date, three generations of EGFR tyrosine kinase inhibitors (TKIs) are clinically available [92]. First-generation EGFR TKIs, including gefitinib and erlotinib, are reversible inhibitors that bind to EGFR and its co-receptor HER2 non-covalently [93], whereas second-generation EGFR TKIs, including afatinib and dacomitinib, bind irreversibly to EGFR [92]. Third-generation EGFR TKIs, such as osimertinib, are also irreversible inhibitors, but show much more efficient blood–brain barrier (BBB) penetration than those from other generations due to its lower efflux by BBB multidrug efflux pumps, suggesting its potential use for treating brain cancer [94,95]. Several phase II clinical studies have shown that osimertinib can be used for treating EGFR-mutant lung adenocarcinoma with brain metastasis [96,97]. Osimertinib shows excellent BBB penetration and significantly inhibits GBM tumorigenesis in vivo [98]. Nevertheless, clinical trials have demonstrated that osimertinib in the treatment of EGFR-mutant GBM was marginally effective due to the intratumoral heterogeneity of GBM [77,99].

Notch1 contributes to GSC maintenance and the modulation of differentiation, and its activity can be effectively blocked by preventing its cleavage using γ-secretase inhibitors [100]. Treatment with the γ-secretase inhibitor, RO4929097, efficiently reduces the viability of the proneural subtype of GSCs, which has a higher gene expression involved in the Notch pathway than the other subtypes [101]. Notch1 positively regulates VEGF activity in GSCs, and patients with high Notch1 expression are more resistant to bevacizumab, an inhibitor of VEGF signaling, indicating that co-inhibition of Notch1 and VEGF might be synergistic [102]. However, a phase I clinical trial of RO4929097 with bevacizumab demonstrated that combination therapy showed little improvement in overall survival (OS) and progression-free survival (PFS) [103]. A separate study showed that the γ-secretase inhibitor, N-[N-(3, 5-difluorophenacetyl-L-alanyl)]-S-phenylglycine t-butyl ester (DAPT), enhances the therapeutic efficacy of TMZ. Treatment with DAPT and TMZ inhibits tumorsphere repopulation and tumor recurrence by suppressing Notch1 signaling [78].

Shh signaling pathway mediates cell proliferation, stem cell fate determination, and differentiation of both normal neural stem cells (NSCs) and GSCs [104,105]. LDE225, a smoothened antagonist that blocks the hedgehog pathway, induces autophagic cell death in GSCs, and CD133-positive cells are more sensitive to LDE225-derived cell death than CD133-negative cells [79]. Moreover, a separate study showed that LDE225 inhibited the expression and nuclear translocation of Gli proteins, which are transcriptional effectors of the Shh signaling pathway [106]. Casein kinase 2 (CK2) is also positively involved in Shh/Gli signaling, and its expression contributes to GSC maintenance via transcriptional activation of β-catenin [107]. An orally bioavailable selective CK2 inhibitor, CX-4945 (silmitasertib), reduces MGMT expression by blocking β-catenin expression in medulloblastoma and sensitized tumor cells to TMZ [108]. In agreement with the results from medulloblastoma, CX-4945 significantly promoted the anti-tumor efficacy of TMZ, both in vitro and in vivo, by downregulating MGMT and pSTAT3 expression levels in GBM [80].

STAT3 is an important regulator of GSCs that is required for cell survival, proliferation, and tumorigenesis in GBM. Both STAT3 inhibitors, STX-0119 and WP1066, suppress GSC proliferation in vitro, but only STX-0119 inhibits tumor growth in a subcutaneous xenograft model of GSCs. Additionally, STX-0119 downregulates the expression levels of GSC markers, including CD44, NANOG, nestin, OLIG2, CD133, and SOX2, as well as STAT3 target genes, including survivin, cyclin D1, c-Myc, MMP9, TGFB1, and VEGF in GSCs [81]. In contrast, WP1066 significantly suppresses intracranial Janus kinase 2 (JAK2)/STAT3 signaling and prolonged the survival of GBM-bearing mice [109]. Napabucasin, a small molecule inhibitor of STAT3, impairs the stemness of GSLCs by inactivating p65/RelA involved in nuclear factor-κB heterodimer formation and suppress the tumor growth in an orthotopic xenograft model [109]. Another small-molecule STAT3 inhibitor, ODZ10117, also decreased the stem cell properties of GSCs and reduced tumor growth in vivo by targeting the SH2 domain of STAT3 [82]. More recently, it was demonstrated that the JAK2-STAT3 signaling pathway is disrupted in GSCs, but bone marrow and X-linked (BMX) non-receptor tyrosine kinase induces STAT3 activation to bypass the suppressor of cytokine signaling 3-mediated negative regulation of JAK2. Consequently, BMX inhibition by ibrutinib specifically disrupts GSCs and suppresses GBM tumor growth, while exhibiting minimal effects on neural progenitor cells activating JAK2-mediated STAT3 [110].

### 3.2. Targeting Autophagy

Although autophagy has both tumor-promoting and tumor-suppressing roles depending on the cellular context, targeting autophagy combined with conventional therapies can be an effective strategy to eliminate GSCs because of its regulatory properties in the stress response [111]. Targeting autophagy related 4B, a key protein in autophagosome formation, with its antagonist (NSC185058) attenuates the tumor-initiating ability of GSCs and sensitizes GBM cells to radiotherapy in orthotopic xenograft mouse models [83]. Chloroquine (CQ) is the most widely used inhibitor of autophagy, which blocks the fusion of autophagosomes with lysosomes, leading to the accumulation of degraded proteins in cells [112]. Currently, four clinical trials have been conducted to test the effectiveness of CQ as an adjuvant treatment for GBM; however, the anti-tumor effects of CQ are not exclusive to GSCs [113]. Combination treatment with radiation, CQ, and PI-103, a dual inhibitor of phosphatidylinositol 3-kinase and mTOR, synergistically induces apoptosis and suppresses tumorsphere formation in GSCs [114]. Similarly, inhibition of autophagy by bafilomycin A1 sensitizes GSCs to radiotherapy and significantly decreases their ability to form tumorspheres [85]. In contrast, adjunctive treatment with quinacrine, an autophagy inhibitor capable of crossing the BBB, enhances the anti-tumor effect of TMZ in primary cultured GSCs, but not in orthotopic xenograft mouse models [86].

### 3.3. Oncolytic Viral Therapy

Oncolytic viral therapy is an emerging novel treatment option for GBM. Oncolytic viral vectors are capable of hijacking cellular machinery or inducing cell lysis by intracellular viral replication in target cells. Although adenovirus does not integrate into the host cell genome, it can be infected with high titers and is less pathogenic to humans than other viruses. To specifically target GSCs, viral vectors incorporate either GSC-specific promoters or modified viral capsids to bind GSC surface markers [115]. The oncolytic adenovirus, Delta24-RGD, is currently under investigation in a phase II clinical trial for GBM treatment [115]. It is selectively replicated in GSCs expressing an abnormal p16INK4/Rb pathway and mediates autophagic cell death of GSCs by induction of endogenous ATG5 [116]. Furthermore, Delta24-RGD–infected GSCs co-cultured with M2 macrophages induced the transition of macrophages toward the M1 phenotype, with an increase in the expression levels of pro-inflammatory genes and cytokines [117]. In addition, treatment with the oncolytic herpes simplex virus, G47Δ, also shifted macrophages to a tumor-detrimental phenotype with the impaired proliferation and self-renewal of GSCs [118]. Although several clinical trials of oncolytic viral therapy have been performed, clinical viral therapy targeting GSCs still has limitations, such as unstable curative effects due to the excessive heterogeneity of GSCs.

### 3.4. Drug Repositioning

Drug repositioning is a new direction in drug discovery that lowers the overall development costs and risk assessments as the safety of the original drug is already verified and its use approved by regulatory institutions. Metformin, a first-line drug for type 2 diabetes mellitus, was tested in a phase I clinical trial as an adjunctive treatment for GBM along with TMZ, and was found to be safe and feasible for newly diagnosed GBM [119]. Metformin preferentially reduces the tumorsphere ability and viability of GSCs by inhibiting Akt activation, whereas differentiated GBM cells are hardly affected [120]. Furthermore, metformin promotes the differentiation of GSCs into non-GSC phenotypes via the activation of the adenosine monophosphate-activated protein kinase–forkhead box O3 axis [121]. Interestingly, another diabetes drug, glimepiride, impairs GSC maintenance and glycolytic flux and confers radiosensitivity to GBM [46]. Together, these studies indicate that repurposed drugs, especially diabetes drugs, can be used for GSC-targeting therapy; however, further research is needed to identify the relationship between diabetes drug-induced metabolic rewiring and stemness of GSCs.

### 3.5. Targeting GSC Niches

GSCs are localized in GSC niches, which have been identified as protective microenvironments in GBM. So far, five types of GSC niches have been identified: peri-vascular, peri-arteriolar, peri-hypoxic, peri-immune, and extracellular matrix. Each niche contains specific cell types that control GSC maintenance by regulating specific molecular mechanisms [122]. In particular, the peri-vascular niche is the most frequently described GSC niche and constitutes abnormal vasculature with friable blood vessels, lack of organization, hypoxia, and impaired BBB due to excessive angiogenesis. Since GSCs produce high levels of angiogenic factors, such as VEGF, and contribute to abnormal vasculature in the perivascular niche [123,124], targeting angiogenic factors may be an ideal therapeutic strategy for targeting GSCs. Bevacizumab, a recombinant humanized monoclonal antibody against VEGF, was approved by FDA for the treatment of recurrent GBM in 2009. Nevertheless, bevacizumab prolonged the progression-free survival, but did not significantly improve the overall survival in newly diagnosed and recurrent GBM cases [125]. In contrast, in a separate phase III clinical trial, bevacizumab was shown to provide a significant overall survival benefit for patients with isocitrate dehydrogenase 1 wild-type proneural GBM in combination with radiotherapy plus TMZ, indicating that the antiangiogenic therapy for GBM may be subtype-specific [126]. The VEGF receptor (VEGFR), especially VEGFR2, is involved in the survival, proliferation, and migration of GBM, and is also an important target for GBM antiangiogenic therapy [127,128]. VEGFR2 is preferentially expressed in GSLCs and is necessary for vascularization and tumorigenesis by GSLCs [128]. To date, several multikinase VEGFR inhibitors have been developed, and some of them have been evaluated in clinical trials for GBM [129].

## 4. Conclusions

GBM remains an incurable disease owing to little progress in the development of effective therapies. GSCs are responsible for tumorigenesis, therapeutic resistance, and tumor recurrence, but GSC-targeting drugs are not yet used in clinical practice. In this review, we summarized various molecular mechanisms of chemoresistance in GSCs. We also discussed the recent strategies to target GSCs with respect to GSC-specific molecular mechanisms and novel therapeutic approaches, from basic to clinical levels. Overall, the therapeutic strategies described in this review have shown high efficacy in targeting GSCs. In particular, CAR T cell therapy, which can be customized for each individual patient, is currently considered the most promising strategy. However, most have failed to be approved for clinical application because of a lack of understanding of the underlying mechanisms or failure to consider individual characteristics. Hence, further investigation of the resistance mechanisms and clinical research on the subtype-specific pathways of GSCs may offer new directions for GBM therapy.

## Figures and Tables

**Table 1:** Therapeutic compounds targeting GSCs.

Target	Compound	Phase	NCT ID	Disease	Reference
CD133	RW03 CAR-T	N/A	N/A	GBM	[71]
EGFR	Gefitinib	Phase II	NCT00052208	Newly diagnosed GBM	[72]
			NCT00250887	Recurrent GBM	[73]
	Erlotinib	Phase II	NCT00525525	Newly diagnosed GBM	[74]
			NCT00445588	Newly diagnosed GBM and Recurrent GBM	[75]
	Afatinib	Phase II	NCT00727506	Recurrent GBM	[76]
	Dacomitinib	Phase II	NCT01520870	Recurrent GBM	[76]
	Osimertinib	N/A	N/A	GBM	[77]
Notch1	RO4929097	Phase II	NCT01122901	Newly diagnosed GBM and Recurrent GBM	N/A
	DAPT	N/A	N/A	GBM	[78]
Shh pathway	Sonidegib	N/A	N/A	GBM	[79]
	Silmitasertib	N/A	N/A	GBM	[80]
STAT3	STX-0119	N/A	N/A	Recurrent GBM	[81]
	WP1066	Phase I	NCT01904123	Newly diagnosed GBM and Recurrent GBM	N/A
	Napabucasin	Phase I/II	NCT02315534	Newly diagnosed GBM and Recurrent GBM	N/A
	ODZ10117	N/A	N/A	GBM	[82]
Autophagy	NSC185058	N/A	N/A	GBM	[83]
	Chloroquine	Phase III	NCT00224978	Newly diagnosed GBM and Recurrent GBM	[84]
	Bafilomycin A1	N/A	N/A	GBM	[85]
	Quinacrine	N/A	N/A	GBM	[86]
VEGF	Bevacizumab	Approved		Recurrent GBM	[87]
